# Perceptions of Canadian vascular surgeons toward artificial intelligence and machine learning

**DOI:** 10.1016/j.jvscit.2022.06.018

**Published:** 2022-07-19

**Authors:** Ben Li, Charles de Mestral, Muhammad Mamdani, Mohammed Al-Omran

**Affiliations:** a Department of Surgery, University of Toronto, Toronto, ON, Canada; b Division of Vascular Surgery, St. Michael’s Hospital, Unity Health Toronto, Toronto, ON, Canada; c Temerty Centre for Artificial Intelligence Research and Education in Medicine, University of Toronto, Toronto, ON, Canada; d Li Ka Shing Knowledge Institute, St. Michael’s Hospital, Unity Health Toronto, Toronto, ON, Canada; e Institute of Health Policy, Management and Evaluation, Dalla Lana School of Public Health, University of Toronto, Toronto, ON, Canada; f Leslie Dan Faculty of Pharmacy, University of Toronto, Toronto, ON, Canada; g Institute of Medical Science, University of Toronto, Toronto, ON, Canada; h Department of Surgery, King Faisal Specialist Hospital and Research Center, Riyadh, Kingdom of Saudi Arabia

**Keywords:** Artificial intelligence, Machine learning, Perceptions, Survey, Vascular surgery

## Abstract

**Background:**

Artificial intelligence (AI) and machine learning (ML) are rapidly advancing fields with increasing utility in health care. We conducted a survey to determine the perceptions of Canadian vascular surgeons toward AI/ML.

**Methods:**

An online questionnaire was distributed to 162 members of the Canadian Society for Vascular Surgery. Self-reported knowledge, attitudes, and perceptions with respect to potential applications, limitations, and facilitators of AI/ML were assessed.

**Results:**

Overall, 50 of the 162 Canadian vascular surgeons (31%) responded to the survey. Most respondents were aged 30 to 59 years (72%), male (80%), and White (67%) and practiced in academic settings (72%). One half of the participants reported that their knowledge of AI/ML was poor or very poor. Most were excited or very excited about AI/ML (66%) and were interested or very interested in learning more about the field (83.7%). The respondents believed that AI/ML would be useful or very useful for diagnosis (62%), prognosis (72%), patient selection (56%), image analysis (64%), intraoperative guidance (52%), research (88%), and education (80%). The limitations that the participants were most concerned about were errors leading to patient harm (42%), bias based on patient demographics (42%), and lack of clinician knowledge and skills in AI/ML (40%). Most were not concerned or were mildly concerned about job replacement (86%). The factors that were most important to encouraging clinicians to use AI/ML models were improvements in efficiency (88%), accurate predictions (84%), and ease of use (84%). The comments from respondents focused on the pressing need for the implementation of AI/ML in vascular surgery owing to the potential to improve care delivery.

**Conclusions:**

Canadian vascular surgeons have positive views on AI/ML and believe this technology can be applied to multiple aspects of the specialty to improve patient care, research, and education. Current self-reported knowledge is poor, although interest was expressed in learning more about the field. The facilitators and barriers to the effective use of AI/ML identified in the present study can guide future development of these tools in vascular surgery.

Machine learning (ML) is a rapidly advancing field of artificial intelligence (AI) that enables computer technology to learn from data and make predictions without explicit programming.^1^ Specifically, ML leverages advanced technology to model complex relationships between user inputs (eg, patient characteristics, radiologic images) and outputs (eg, disease diagnosis, outcomes).^1^ An automated algorithm can then be built that will learn from large amounts of data to make predictions that allow clinicians to better understand the future clinical course of patients, guiding critical management decisions.^1^ The field has been driven by the explosion of electronic data combined with increasing computational power.^1^ In vascular surgery, AI/ML algorithms have been developed to predict abdominal aortic aneurysm growth,^2^ detect endoleaks,^3^ and identify patients with peripheral artery disease who have a high mortality risk.^4^ Despite an increasing amount of research interest in AI/ML techniques, its translation to real-world practice has remained limited.^5^

The knowledge, attitudes, and behaviors of vascular surgeons regarding AI/ML will have important effects on the application of these technologies to clinical practice. Previous studies have assessed the perceptions of clinicians toward AI/ML in pathology,^6^ psychiatry,^7^ and primary care.^8^ These studies have demonstrated that physicians are excited by the potential of AI/ML to improve outcomes and efficiency but are also concerned about the ethical, legal, and patient safety implications of these tools. Given the recent advances in AI/ML technologies and their potential to transform clinical practice, we conducted a survey of Canadian vascular surgeons to better understand their knowledge, attitudes, and perceptions regarding AI/ML technology.

## Methods

### Ethics approval

The Unity Health Toronto research ethics board approved the present study. All the participants provided written informed consent. The survey responses were kept anonymous, and no personal identifying information was collected.

### Study design and participants

A survey of Canadian vascular surgeons regarding their perceptions of AI/ML was conducted and reported in accordance with the Checklist for Reporting Results of Internet E-Surveys.^9^ We distributed a self-administered online questionnaire to members of the Canadian Society for Vascular Surgery through a national e-mail list using Google Forms.^10^ The questionnaire was sent on January 17, 2022, with two follow-up reminders, and closed on February 21, 2022. Completion of the survey was voluntary, and the participants did not need to respond to every question. They could also change their answers throughout the survey up until submission. We used the Google Forms function to allow only one response per participant. Respondents received a preamble with background information and definitions regarding AI/ML before completing the questionnaire (Appendix 1).

### Survey design

The survey contained 11 questions that requested information from participants regarding their demographics and knowledge, attitudes, and perceptions regarding AI/ML technology (Appendix 2). The demographic data collected included age, gender, self-reported race, and practice setting (academic vs nonacademic). Using a 5-point Likert scale, we assessed their self-reported knowledge of AI/ML (from 1, very poor; to 5, very good), attitudes toward the incorporation of AI/ML into vascular surgery (from 1, very concerned; to 5, very excited), and willingness to learn more about the technology (from 1, no interest; to 5, very interested). Similarly, we assessed the participants’ perceptions of AI/ML in terms of (1) the usefulness of potential applications in various areas of vascular surgery (ie, diagnosis, prognosis, patient selection, image analysis, intraoperative guidance, research, education; from 1, not useful; to 5, very useful); (2) concerns regarding limitations (ie, errors, patient privacy, inadequate clinician knowledge, patient discomfort, bias, lack of trust, job replacement; from 1, not concerned; to 5, very concerned); and (3) the importance of facilitators (ie, better diagnostic or predictive accuracy compared with clinicians, validation using specific patient populations, simple to understand and easy to use, improvement in efficiency, adequate trust, not biased; from 1, not important; to 5, very important). These factors were generated by us from previous literature on AI/ML applications,^11^, ^12^, ^13^, ^14^ limitations,^5^^,^^15^, ^16^, ^17^, ^18^ and facilitators.^19^, ^20^, ^21^, ^22^ The questions were modeled on previous surveys of clinicians’ perceptions of AI/ML in other fields of medicine.^6^, ^7^, ^8^^,^^23^, ^24^, ^25^ At the final question, the respondents could include additional free text on topics they believed were relevant.

### Statistical analysis

The survey results were analyzed in aggregate and are expressed as numbers and proportions. The respondent demographics were reported categorically: age (≤29, 30-39, 40-49, 50-59, ≥60 years), gender (male, female, other), race (Asian, Black, Hispanic, White, other), and practice setting (academic, nonacademic). The participants’ self-reported knowledge, excitement about AI/ML, and interest in learning more about the field are reported in tabular format. The perceptions of AI/ML applications, limitations, and facilitators are graphically represented. All statistical analyses were performed using R, version 4.1.2 (R Foundation for Statistical Computing, Vienna, Austria).^26^

## Results

### Respondent characteristics

Overall, 50 of the 162 Canadian vascular surgeons (31%) responded to the survey. Most respondents were aged 30 to 59 years (72%), male (80%), and White (67.4%) and practiced in an academic setting (72%; Table I).

**Table I:** Respondent characteristics (total, N = 50)

Characteristic	Respondents, No. (%)
Age, years	
≤29	4 (8)
30-39	10 (20)
40-49	12 (24)
50-59	14 (28)
≥60	10 (20)
Gender	
Male	40 (80)
Female	10 (20)
Race	
Asian	9 (19.6)
Black	1 (2.2)
Hispanic	1 (2.2)
White	31 (67.4)
Other^a^	8 (16.0)
Practice setting	
Academic	36 (72)
Nonacademic	14 (28)

a Self-reported and included Middle Eastern, Turkish, Arabic, and not reported.

### Self-reported knowledge, attitudes, and perceptions of AI/ML

One half of the respondents (50%) reported their knowledge of AI/ML as poor or very poor. Most participants (66%) were excited or very excited about the incorporation of AI/ML into vascular surgery. Finally, most (84%) were interested or very interested in learning more about AI/ML (Table II).

**Table II:** Canadian vascular surgeons’ (total, N = 50) self-reported knowledge, attitude, and perceptions of artificial intelligence (AI) and machine learning (ML)

Question and response	Participants, No. (%)
How would you rate your knowledge of AI/ML?	
Very poor	8 (16)
Poor	17 (34)
Average	14 (28)
Good	9 (18)
Very good	2 (4)
How do you feel about the incorporation of AI/ML into vascular surgery?	
Very concerned	0 (0)
Concerned	1 (2)
Neutral	16 (32)
Excited	20 (40)
Very excited	13 (26)
Are you interested in learning more about AI/ML?	
No interest	1 (2)
Little interest	1 (2)
Neutral	6 (12.2)
Interested	17 (34.7)
Very interested	24 (49.0)

### Potential applications of AI/ML in vascular surgery

**Fig 1:**
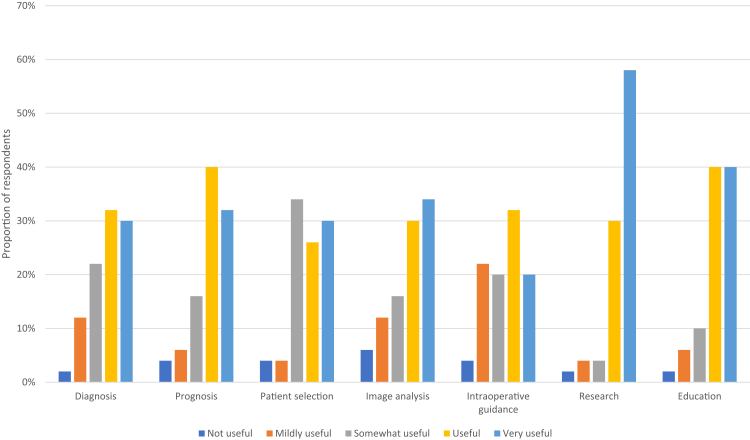
Participants’ response to the survey question: for each of the following areas in vascular surgery, how useful would artificial intelligence (AI)/machine learning (ML) be (scale from 1 [not useful] to 5 [very useful])?

Most respondents believed that AI/ML would be useful or very useful in vascular surgery regarding diagnosis (62%), prognosis (72%), patient selection (56%), image analysis (64%), and intraoperative guidance (52%). The areas that most participants had rated as useful or very useful were research (88%) and education (80%; Fig 1).

### Concerns about AI/ML

**Fig 2:**
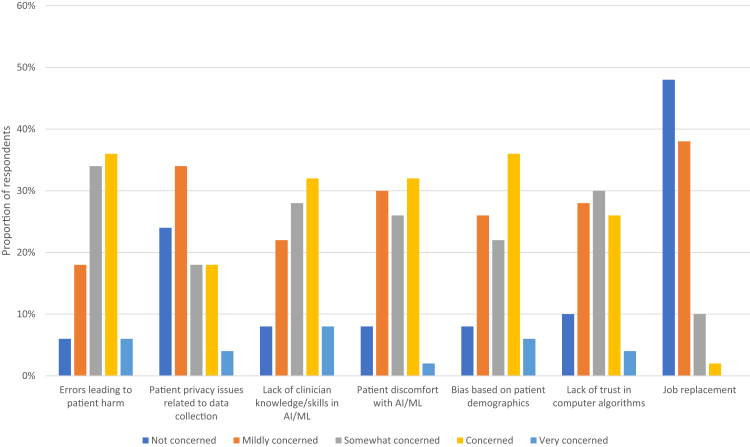
Participants’ response to the survey question: how concerned are you about each of the following limitations of artificial intelligence (AI)/machine learning (ML; scale from 1 [not concerned] to 5 [very concerned])?

The areas of AI/ML for which most participants indicated they were concerned or very concerned were errors leading to patient harm (42%), bias based on patient demographics (42%), and lack of clinician knowledge and/or skills in AI/ML (40%). Most respondents were not concerned or mildly concerned about job replacement (86%; Fig 2).

### Facilitators of AI/ML

**Fig 3:**
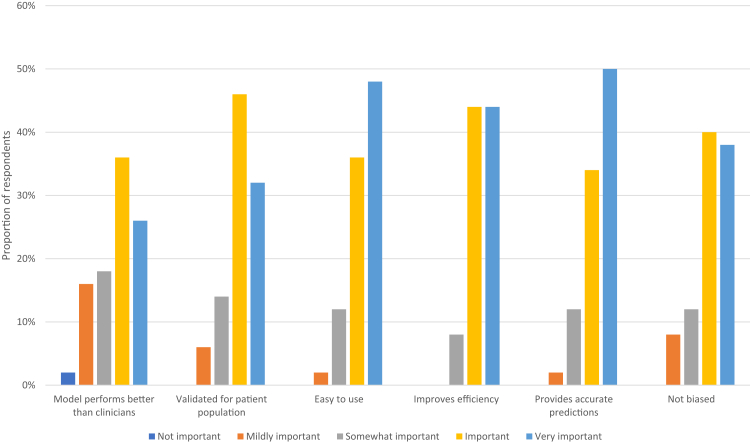
Participants’ response to the survey question: how important are each of the following factors in encouraging you to use an artificial intelligence (AI)/machine learning (ML) model (from 1 [not important] to 5 [very important])?

Most respondents believed that the following aspects were important or very important to encouraging them to use an AI/ML model: improves efficiency (88%), provides accurate predictions (84%), easy to use (84%), validated for a patient population (78%), not biased (78%), and model performs better than clinicians (62%; Fig 3).

### Additional comments regarding AI/ML from respondents

The following are selected direct quotes from the participants: “Very exciting field with lots of potential to improve patient care”; “I feel this is an important step towards improving vascular care”; “What took us so long? We are lagging behind compared to other specialties”; and “Let’s develop these tools to make things better.”

## Discussion

### Summary of findings

The results from our national survey have demonstrated that Canadian vascular surgeons have positive views about AI/ML and its potential to improve patient care, research, and education. Their current self-reported knowledge is poor, and most participants were willing to learn more about this technology. The respondents indicated that the limitations of AI/ML they were most concerned about were errors leading to patient harm, model bias, and their lack of knowledge and/or skills to effectively use AI/ML tools. Important facilitators that would encourage the use of AI/ML in clinical practice were improvement of efficiency, ability to provide accurate predictions, and ease of use.

### Comparison to existing literature

To the best of our knowledge, our survey is the first to assess the perceptions of vascular surgeons toward AI/ML. Surveys of clinicians in other specialties have been previously conducted. In 2019, Sarwar et al^6^ assessed physician perspectives on the integration of AI into diagnostic pathology. They demonstrated that 75% of pathologists reported interest or excitement in AI and that 80.4% believed that this technology would not significantly affect the job market.^6^ In 2020, Doraiswamy et al^7^ performed a global survey of psychiatrists and found that 50% believed that their jobs would be substantially changed by AI/ML, which would be especially useful regarding updating medical records and synthesizing information. A qualitative analysis of primary care providers by Blease et al^8^ demonstrated that the perceived benefits of AI/ML were improvements in efficiency and a reduction of administrative burdens. However, the respondents expressed concerns regarding the acceptability of AI/ML to providers and patients owing to the social and ethical implications of artificial technology in medicine.^8^ Castagno and Khalifa^23^ surveyed clinicians across multiple specialties and found that 79% believed that AI could be useful or extremely useful in their specialty but that knowledge was poor, with only 13% knowing the difference between machine learning and deep learning. We have demonstrated similar findings in our survey of vascular surgeons, with most respondents indicating excitement about potential AI/ML applications, understanding about the limitations of the technology, and recognition of their poor current knowledge with a willingness to receive further education in the field.

AI/ML has been applied to other disciplines in a variety of ways. In neurosurgery, ML algorithms have been developed to predict survival, recurrence, and adverse events for patients undergoing surgery for malignancies, spinal lesions, and traumatic brain injury, with a median accuracy of 94.5%.^27^ In radiology, ML has been applied to medical image segmentation, computer-aided diagnosis, and text analysis of radiology reports through natural language processing, identifying complex patterns automatically and helping radiologists make decisions more effectively and efficiently.^28^ Our group recently reported a systematic review of ML applications in vascular surgery and identified 212 studies using ML techniques for diagnosis, prognosis, and image segmentation in carotid stenosis, aortic aneurysm/dissection, peripheral artery disease, diabetic foot ulcer, venous disease, and renal artery stenosis.^29^ AI/ML can be used to achieve the goals of improving efficiency in clinical practice by learning from large amounts of data to make automated predictions to guide clinical decision-making.^30^ The advantage of using ML models is that they can quickly analyze large amounts of data, including a patient’s demographic information, medical history, previous clinical encounters, and imaging data.^30^ Given the increasing demands on busy clinicians who might not have the time to process all this information systematically, ML algorithms can improve efficiency and effectiveness of clinical decision-making.^31^ ML can also be used in the medical education process in a variety of ways. One example would be to offer real-time surgical simulation by training neural networks on large amounts of physics-based computational data, emulating the surgical environment, including human tissue, instruments, and tactile responses.^32^ This would allow trainees to practice effectively before providing direct patient care in the operating room.^32^

### Explanation of findings

Several explanations are possible for our results. First, the respondents indicated that AI/ML would be useful when applied to various aspects of vascular surgery, including diagnosis, prognosis, patient selection, image analysis, intraoperative guidance, research, and education. This is supported by existing literature demonstrating that AI/ML algorithms can be trained to diagnose peripheral artery disease^4^ and predict mortality risk,^33^ analyze imaging data to predict abdominal aortic aneurysm growth,^2^ automate article screening for systematic reviews,^34^ and facilitate surgical simulations.^32^ A systematic review of ML applications in vascular surgery included 212 relevant studies on diagnosis, prognosis, and image segmentation for six major vascular conditions with good predictive value.^29^ The participants reported that AI/ML would be most useful in research and education, reflecting that this technology remains in the research and development phase, with limited applications in routine clinical settings. As AI/ML algorithms become deployed clinically, the perceptions of vascular surgeons regarding areas in which the technology could be helpful could evolve. Second, the vascular surgeons indicated that the most significant barriers to the effective use of AI/ML were errors leading to patient harm, bias based on patient demographics, and lack of clinician knowledge and/or skills in AI/ML. Previous work has identified similar concerns, corroborating the need to rigorously evaluate models using representative populations and provide adequate clinician education before deployment.^35^, ^36^, ^37^ The frameworks for developing and implementing successful ML models have been previously described.^38^, ^39^, ^40^, ^41^ The key steps include generating a clinically relevant question, building a collaborative team of clinicians and computer scientists, and evaluating the model for generalizability and biases.^38^, ^39^, ^40^, ^41^ Ensuring that these limitations are carefully considered during model development and evaluation will be critical to the successful deployment of AI/ML in vascular surgery. However, most vascular surgeons were not concerned about job replacement. This supports existing literature suggesting that AI/ML is intended to augment, rather than replace, clinicians.^42^ Third, our participants expressed a sense of urgency regarding the need to implement AI/ML tools in vascular surgery. Given the potential for this technology to improve care delivery and patient outcomes, earlier implementation of rigorously tested models will be beneficial to providers and patients.^43^ Important facilitators identified by clinicians were the need for algorithms to improve efficiency, provide accurate predictions, and be easy to use. Given the increasing workload with an aging population, administrative burdens, and time constraints on clinicians, it is understandable that respondents favor the use of AI/ML models that will improve both the efficiency and the effectiveness with which they provide care.^44^, ^45^, ^46^ The development of future AI/ML tools should focus on these factors to facilitate successful implementation in routine vascular surgical care. Fourth, our survey has demonstrated that vascular surgeons currently rate their knowledge of AI/ML as poor; however, they expressed positivity about the field and want to learn more. This likely reflects the fact that clinicians receive limited teaching in AI/ML during their training, and formal education programs designed for health care providers will be critical to facilitate successful implementation of AI/ML tools.^47^ Incorporation of AI/ML education into the medical school curriculum, open online courses, and hands-on workshops are strategies to improve clinicians’ knowledge and skills in using this technology.^48^^,^^49^

### Study limitations

The present study had several limitations. First, the response rate was 31%. Online surveys are known to generate lower response rates,^50^ especially when directed toward clinicians.^51^ In other fields of medicine, clinician surveys of AI/ML have had response rates that have varied widely from 1.3% to 72%.^23^^,^^52^, ^53^, ^54^ Our response rate is consistent with those reported for other surveys of Canadian vascular surgeons.^55^^,^^56^ Second, most of the respondents were White men practicing in academic settings, which might not adequately represent the perspectives of women and non-White populations working in nonacademic settings. These demographics reflect the current vascular surgery workforce,^57^ as demonstrated in previously reported surveys of Canadian vascular surgeons.^55^^,^^56^^,^^58^ Similar demographic distributions are present in the U.S. vascular surgery workforce.^59^ We would recommend conducting an updated survey in the future as increasing efforts are underway to improve workforce diversity.^60^ Third, we provided an option for respondents to input free-text comments; however, a qualitative method was not applied to obtain more in-depth perspectives. Future qualitative studies using one-on-one interviews or focus groups might provide greater insight into the perceptions of vascular surgeons toward AI/ML. Fourth, we did not include questions on cost-effectiveness and the effects on personal efficiency in the survey. Future studies could include these topics to further strengthen our understanding of vascular surgeons’ views of AI/ML technology.

## Conclusions

In the present survey of Canadian vascular surgeons, we have demonstrated that respondents have positive views about AI/ML and its potential to improve patient care, research, and education. The facilitators and barriers identified in the present study can guide the future development of clinically relevant AI/ML tools in vascular surgery that consider the perspectives of end users. The current self-reported knowledge is poor, and most vascular surgeons are willing to learn more about the field. Opportunities exist to improve AI/ML knowledge in vascular surgery through formal education programs.
