# Supplementary material for: Perceptions of Canadian vascular surgeons toward artificial intelligence and machine learning
Source: J Vasc Surg Cases Innov Tech. 2022 Jul 19;8(3):466–72. doi: 10.1016/j.jvscit.2022.06.018 (PMC9396444; doi:10.1016/j.jvscit.2022.06.018)
Supplement: Supplement [file mmc1.docx]

**Appendix 1: Preamble**

Artificial intelligence (AI) is a rapidly advancing field of computer science whereby systems are designed to simulate intelligent behaviour and critical thinking comparable to humans. Machine learning (ML) is a subfield of AI that enables computers to learn from data to identify patterns and make predictions without explicit programming. Examples of ML tools include neural networks, deep learning, and natural language processing. The value of AI/ML is that these technologies can automatically and quickly analyze large amounts of data to augment a clinician’s ability to diagnose disease and make predictions about a patient’s clinical course, among other applications.

In vascular surgery, AI/ML algorithms have been applied to predict abdominal aortic aneurysm growth (<https://pubmed.ncbi.nlm.nih.gov/29988820/>), detect endoleaks (<https://www.nature.com/articles/s41598-020-74936-7>), and identify patients with peripheral artery disease who have high mortality risk (<https://pubmed.ncbi.nlm.nih.gov/27266594/>).

For more background information on AI/ML, please refer to the following article (<https://www.ncbi.nlm.nih.gov/pmc/articles/PMC6616181/>).

Given recent advances in AI/ML and its potential to transform health care, it is important to understand your perceptions towards these technologies with regards to clinical decision making. This questionnaire takes approximately 5 minutes or less to complete.

**Appendix 2: Survey**

1. What is your age?

a) ≤ 29

b) 30 – 39

c) 40 – 49

d) 50 – 59

e) ≥ 60

2. What is your gender?

a) Male

b) Female

c) Not listed (please specify)

3. What is your race?

a) Asian (including East and South Asian)

b) Black

c) Hispanic

d) White

e) Not listed (please specify)

4. What is your practice setting?

a) Academic

b) Non-academic

5. How would you rate your knowledge of artificial intelligence (AI) and machine learning (ML)?

a) Very poor

b) Poor

c) Average

d) Good

e) Very good

6. For each of the following areas in vascular surgery, how useful would AI/ML be (scale of 1 [not useful] to 5 [very useful])?

a) Diagnosis (e.g. identifying endoleak on imaging)

b) Prognosis (e.g. predicting mortality following surgery)

c) Patient selection (e.g. assessing risk of post-operative complications to inform risk/benefit discussions with patients)

d) Image analysis (e.g. outlining atherosclerotic plaque on imaging)

e) Intraoperative guidance (e.g. identifying high risk actions using video technology)

f) Research (e.g. automating data collection/analysis)

g) Education (e.g. surgical simulations)

7. How concerned are you about each of the following limitations of AI/ML (scale of 1 [not concerned] to 5 [very concerned])?

a) AI/ML algorithms may make errors that harm patients

b) The amount of data required to develop AI/ML algorithms may lead to patient privacy issues

c) Clinicians may not have the knowledge or skills to use AI/ML effectively

d) Patients may not be comfortable with the application of AI/ML to their care

e) AI/ML algorithms can be biased towards specific populations based on demographics (e.g. age, sex, and race)

f) Computer algorithms cannot be trusted to make clinical decisions

g) AI/ML will reduce the demand for vascular surgeons and replace jobs

8. How important are each of the following factors in encouraging you to use an AI/ML model (1 [not important] to 5 [very important])?

a) The model has been demonstrated to perform better than clinicians in terms of diagnostic or predictive accuracy

b) The model has been validated for my patient population

c) The model is simple to understand and easy to use

d) The model improves my efficiency in providing effective clinical care

e) I can trust the model to provide accurate predictions

f) The model is not biased towards a specific population based on demographics (e.g. age, sex, and race)

9. How do you feel about the incorporation of AI/ML into vascular surgery?

a) Very concerned

b) Concerned

c) Neutral

d) Excited

e) Very excited

10. Are you interested in learning more about AI/ML?

a) Very interested

b) Interested

c) Neutral

d) Little interest

e) No interest

11. Please provide any additional comments you have regarding AI/ML in vascular surgery.
